# Pollution and risk assessment of heavy metals in rivers in the antimony capital of Xikuangshan

**DOI:** 10.1038/s41598-022-18584-z

**Published:** 2022-08-23

**Authors:** Qing Xie, Bozhi Ren

**Affiliations:** School of Resources, Environment and Safe Engineering, Hunan University of Science and Technology, Xiangtan, 411201 Hunan China

**Keywords:** Biochemistry, Environmental sciences, Risk factors

## Abstract

Xikuangshan (XKS) is the world's largest antimony mining region, and its exploitation for hundreds of years has also resulted in serious soil erosion, fragile ecology, contaminated water, and shortage water. Through systematic and scientific collection samples from the rivers in XKS, the Nemerow index (NI), modified heavy metal pollution index (m-HPI), ecological risk index, and health risk indexeswere used to evaluate and analyze the water quality, pollution levels and risks of heavy metals (Sb, As, Mn, Pb, Zn, Hg, Cd) to ecology and humans in XKS. The results showed that the average concentrations of TN, TP, Sb, As and Hg in surface water were 0.48 mg/L (0–4.34 mg/L), 2.58 mg/L (0–4.34 mg/L), 1.05 mg/L (0.0009–5.33 mg/L), 1.06 mg/L (BDL–19.60 mg/L) and 0.00084 mg/L (LDBL–0.0036 mg/L), respectively, exceeding the limits of the Chinese surface water quality standards. Based on the m-HPI method, only 8.57% of the sampling points are classified as the worst water quality. However, according to the NI method, about 7.14% and 87.16% of the sampling points in the study area are moderately and severely polluted, respectively. The results of heavy metal pollution based on the NI evaluation is were more serious than that on the m-HPI method. The values of ecological risk assessment varied from 22.69 to 7351.20, revealed that heavy metals pose a very serious risk to the surface water ecosystem at more than 50% of the sampling sites, and Sb and As are the main pollutants, followed by Hg. The total non-carcinogenic risk index (TCR) for adults and children were 47.70 and 90.10 respectively, Sb and As is the main non-carcinogenic risk factor. For adults and children, the average carcinogenic risk (CR) of As was 6.49 × 10^–3^ and 1.05 × 10^–2^, respectively, and exceeded the threshold of 1 × 10–^4^, indicating a high carcinogenic risk.

## Introduction

Water plays an important role in ecological systems, and is an indispensable component of human lives. As open environmental spaces, rivers are more likely to be polluted^1^. This issue has received a great deal of attention, because concentrations of heavy metals in rivers continued to rise above the regional background value under constant anthropogenic disturbances, and has caused potential impacts on human health and the ecological balance^2–6^. Heavy metals in watershed ecosystems are difficult to be degrade and characterized by strong concealment, resulting a lasting harm and difficult to removal^7^. They exhibited different degrees of risk, impacts by point and non-point surface pollution, and characteristics of bioaccumulation and anthropogenic dominant sources^4^. Most studies have focused on lakes, river basins, and bays^3,4,7,8^, and few have been conducted from the perspective of water bodies in mining areas.

Antimony (Sb) is the ninth most mined industrial metal in the world, and Sb and its compounds are considered to be emerging pollutants^9^. As the largest antimony mining area in the world, the Xikuangshan area (XKS) has hosted hundreds of antimony mining, processing, and smelting enterprises throughout history, and also produced a great deal of exposed waste residue disposal sites. The main mineral in the deposit is stibnite (Sb_2_S_3_), with trace amounts of pyrite (FeS_2_), pyrrhotite (Fel-xS), and sphalerite (ZnS)^10^. Many studies have been conducted on the spatial distribution, migration characteristics, bioavailability and risk assessment of soil heavy metals from XKS in recent years^11–17^, but researches on heavy metal pollution in water bodies were few, and only focused on the Sb and/or As elements^4,11,14,18^. Pollution of Sb and its associated metals in water bodies has seldom been studied.

Therefore, the goals of this study were (1) to systematically investigate the distributions of Sb and its associated elements in the surface water in XKS; (2) to assess the levels of the pollution using both the Nemerow multi-factor index (NI) and the modified heavy metal pollution index (m-HPI); (3) to use the ecological risk index (ERI) to assess the ecological hazards; and (4) to assess the health risks of heavy metals to adults and children through direct ingestion and skin contact. The results of this study provide a basis for the precise remediation of regional watersheds pollution and the protection of positive cycle of the ecological environmental in mining areas.

## Materials and methods

### Study area

**Figure 1:**
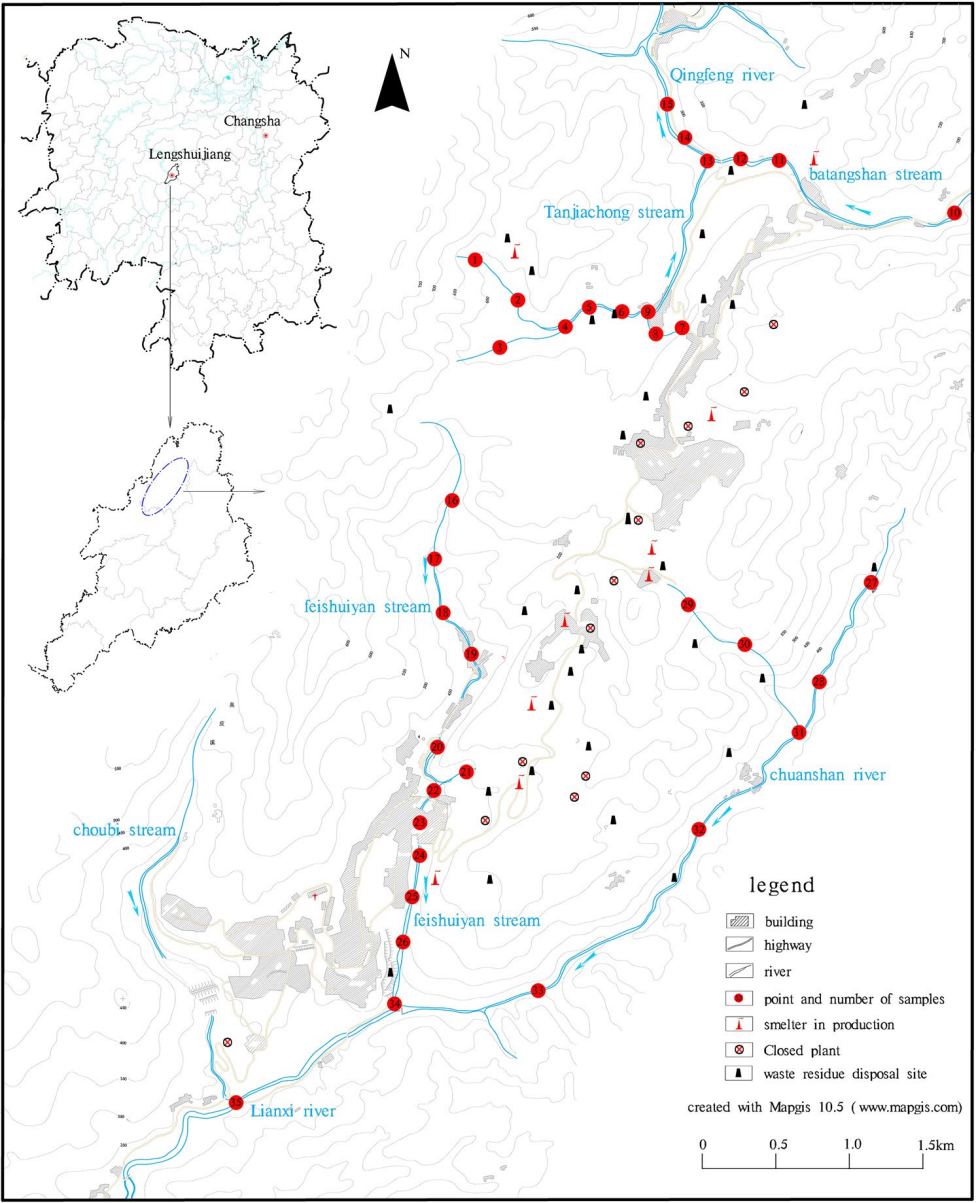
Distribution of sampling points in the XKS area.

The XKS is located in Lengshuijiang city, Hunan Province, China, with a total area of 70 km^2^ and an altitude of 200–400 m^15^. The terrain is high in the north and low in the south. This area has a subtropical continental monsoon climate, with an average annual temperature of 16.7 °C and an annual rainfall of 1354 mm^11^. The river system in this area is well developed, including the Qingfeng (QF) River and the Lianxi (LX) River (Fig. 1). The Qingfeng River runs through the north part of the XKS, where the Tanjiachong (TJC) stream and the Batangshan (BT) stream converge. The Lianxi River is located in the south part of the XKS, and it can be divided into upstream (Chuanshan creek (CS) and Feishuiyan (FSY)) and downstream (Lianxi River) components.

### Sampling and analysis

In order to evaluate the changes in the heavy metal concentrations in the surface water of the XKS area, a total of 35 samples with a depth of 0–5 cm were collected from the rivers in May 2020 according to the Technical Specifications Requirements for Monitoring of Surface Water and Waste Water (HJ/T91-2002) (Fig. 1). And 15 water samples were collected in QF river, including 9 samples in TJC tream (TJC-1–9), 3 samples in BT Stream (BT-10–12), and 3 samples in the confluence and downstream (QF-13–15). A total of 20 samples were collected in the LX river, including 11 samples from FSY creek (FSY-16–26), 7 samples from CS creek (CS-27–33) and 2 samples from the intersection and downstream (LX34–35). To ensure the location of sampling point are accurate, portable global positioning systems (GPS) were used. All samples were filtered using a pre-weighted fiberglass filter (0.45 um) to remove suspended particles, then stored in sealed containers at temperatures below 5 °C and shipped back to the laboratory.

Based on the monitoring data from the local environmental monitoring stations, as well as the local residents’ habits regarding domestic water and agricultural irrigation, the pH value, potassium permanganate index (CODMII), chemical oxygen demand (COD), five-day biochemical oxygen demand (BOD_5_), ammonia nitrogen (NH_3_–N), total phosphorus (TP), total nitrogen (TN), and heavy metal contents (Sb, As, Mn, Pb, Zn, Hg, and Cd) were determined. A Phs-3c pH meter was used to measure the pH value of the surface water. The CODMII of the water was determined using the acidic potassium permanganate method (GB 11892-1989). The COD was determined using dichromate method (HJ 828-2017), and the BOD_5_ was determined using dilution and inoculation tests (HJ 505-2009). The NH_3_-N, TP, and TN concentration were determined using Nessler's reagent colorimetric method, the ammonium molybdate spectrophotometric method, and alkaline potassium persulfate digestion UV spectrophotometry method, respectively. The Sb, As and Hg contents were determined using the atomic fluorescence method (AFS 8220), Mn and Zn contents determined using flame atomic absorption spectrophotometry (TAS-990AFG), and the Pb and Cd contents were determined using graphite atomic absorption spectrophotometry (AA240Z).

Throughout the sample collection, transportation, storage and testing processes, all of the operations were carried out in accordance with the Technical Specifications Requirements for Monitoring of Surface Water and Waste Water (HJ/T91-2002). The entire quality control process was implemented using 20% quality control samples and 10% parallel samples to ensure the precision, accuracy, and reproducibility of sample determination of the data.

### Heavy metal pollution in surface water

#### Modified heavy metal pollution index

The m-HPI method^19^ is proposed based on the heavy metal pollution index (HPI)^20^ and the heavy metal evaluation index (HEI)^21^. The HPI method takes the highest desirable concentration (MDL) and the maximum permissible concentration (MPL) of the different metals into consideration, while the HEI method uses the maximum allowable concentrations (MAC) of the metals to characterize the water quality. However, due to the update of the heavy metal supervised guideline, the categories of some metals have been redefined. Thus, the methods in the reference the previous guidelines have some limitations when applied in some fields, but the m-HPI does not^5^.

This m-HPI method uses the positive index (PI) and negative index (NI) to evaluate the water quality^5^. The PI reflects the contribution of metals to the water quality when the metal concentrations are lower than the threshold values. And when the PI values of two water samples are similar, the NI can further classify the quality grades. The m-HPI is calculated as follows:

$$ {\text{NI}} = \left\{ {\begin{array}{*{20}c} {0,} & {{\text{when}}\; n_{1} = 0} \\ {\mathop \sum \limits_{i = 1}^{{n_{1} }} {\text{mHPI}}_{i} } & {n_{1} > 0} \\ { - 1,} & {{\text{when}} \;M_{i} = {\text{DBL}}} \\ \end{array} } \right. $$

$$ PI = \left\{ {\begin{array}{*{20}c} {0,} & {{\text{when}} \;n_{2} = 0} \\ {\mathop \sum \limits_{i = 1}^{{n_{1} }} {\text{mHPI}}_{i} } & {n_{2} > 0} \\ {0,} & {{\text{when}} M_{i} = DBL } \\ \end{array} } \right. $$

where, ${\text{mHPI}}^{i}$ is the modified heavy metal pollution index, n is the number of total metals (*n* = 7), and n_1_ and n_2_ are to be treated as independent and mutually exclusive subsets such that *n* = *n*_1_ + *n*_2_. DBL denotes ‘below the detection limit’.

$$ {\text{m - HPI}}^{i} = \omega_{i} Q_{i} $$

$$ \omega_{i} = \frac{{W_{i} }}{{\mathop \sum \nolimits_{i = 1}^{n} W_{i} }} $$

$$ W_{i} = \frac{1}{{I_{i} }} $$

$$ Q_{i} = \frac{{M_{i} - I_{i} }}{{I_{i} }} $$

$\omega_{i}$ is the relative weight of a factor, and is inversely proportional to the highest desirable concentration ($I_{i}$), $M_{i}$ is the measured concentration of metal $i$, and $I_{i}$ refers to the Chinese drinking water standard^22^ and the maximum allowable concentration of the World Health Organization (WHO) standard^23^. $Q_{i}$ is not a modulus, and it will be zero when $M_{i}$ < $I_{i}$, which means the samples pass the quality standard based on a specific parameter. When $M_{i}$ > $I_{i}$, $Q_{i}$ will be positive. For all of the metals $M_{i}$ = $S_{i}$, the upper limit of PI (U_L_) was calculated to be 54.51. The water quality was classified into four categories^5^: excellent (− 1 ≤ NI ≤ 0, PI = 0), very good (-1 ≤ NI ≤ 0, 0 ≤ PI ≤ UL/2), good (− 1 ≤ NI ≤ 0, UL/2 ≤ PI ≤ UL), and unacceptable (NI ≤ 0, PI > UL).

#### Nemerow index

The Nemerow multi-factor index is a comprehensive evaluation method ^4,24^, and it is calculated as follows:

$$ NI = \left[ {\left\{ {\left( {M_{i} /I_{i} } \right)_{{{\text{mean}}}}^{2} + \left( {M_{i} /I_{i} } \right)_{\max }^{2} } \right\}/n} \right]^{\frac{1}{2}} $$

where, $\left( {M_{i} /I_{i} } \right)_{{{\text{mean}}}}$ and $\left( {M_{i} /I_{i} } \right)_{{{\text{max}}}}$ are the respective mean and maximum values of $\left( {M_{i} /I_{i} } \right)$ for metal $i$ in water. NI can be divided into four grades^4,24^: no pollution (NI < 1), slight pollution (1 ≤ NI < 2.5), moderate pollution (2.5 ≤ NI < 7), and severe pollution (NI ≥ 7).

### Ecological risk

The ERI was used to evaluate the potential ecological risk posed by the heavy metals in the surface water system ^4,25^. It is calculated as follows:

$$ {\text{ERI}} = \mathop \sum \limits_{i = 1}^{n} \left[ {T_{i} \times \left( {M_{i} /I_{i} } \right)} \right] $$

where, $T_{i}$ is the biological toxicity factor of metal $i$ (Sb = 5, As = 10, Mn = 1, Pb = 5, Zn = 1, Hg = 40, and Cd = 30)^25,29^. The ERI is divided into four categories: low risk (ERI < 110), moderate risk (110 ≤ ERI < 200), considerable risk (200 ≤ ERI < 400), and very high risk (ERI ≥ 400).

### Human health risk assessment

Health risk assessment of heavy metals in aquatic environments usually considers two pathways, direct ingestion and dermal contact^4,26,27^. The daily exposure doses of direct ingestion (${\text{ADD}}_{{{\text{ing}}}}$) and dermal absorption (${\text{ADD}}_{{{\text{derm}}}}$) for adults and children are:

$$ {\text{ADD}}_{{{\text{ing}}}} = \frac{{M_{i} \times {\text{IR}} \times {\text{ABS}}_{i} \times {\text{EF}} \times {\text{ED}}}}{{{\text{BW}} \times {\text{AT}}}} $$

$$ {\text{ADD}}_{{{\text{derm}}}} = \frac{{M_{i} \times {\text{SA}} \times {\text{KP}}_{i} \times F \times {\text{EF}} \times {\text{ET}} \times {\text{CF}} \times {\text{ED}}}}{{{\text{BW}} \times {\text{AT}}}} $$

where, $M_{i}$ is the measured concentration of metal $i$ (mg/L); $IR$ is the intake rate (L/day, 2.0 for adults and 0.64 for children). $SA$ is the exposed skin area (in cm^2^) exposed to surface water (18,000 for adults and 6,600 for children); $ET$ is the exposure time (hours/day, 0.58 for adults, 1.00 for children); $ED$ is the duration of exposure (70 years for adults and six years for children). $EF$ is the exposure frequency (days/year), and its value is 350; $BW$ is the bodyweight of the residents (65 kg for adults and 20 kg for children). $AT$ is the average exposure time in days (25,550 days for adults and 2190 days for children). $KP_{i}$ is the dermal permeability coefficient of metal $i$ (Table 1). $ABS_{i}$ is the absorption factor of the stomach and intestines (Table1). $F$ is the ratio of the skin contact surface with water, and its value is 0.9. $CF$ is the volume conversion coefficient for water (1L/1000 cm^3^,10^–3^). All of the parameter values except $M_{i}$ are reference with the related articles ^4,26–28^.

**Table 1:** Regulatory limits in drinking water, biological toxicity factor, gastrointestinal absorption factor of the target heavy metals, and exposure parameters.

Heavy metal	Is carcinogen	T_i_^25,29^	ABS_i_^11,26^	KP^11,26^ (cm/hour)	RfD_ing_^4^ (mg/kg/d)	RfD_der_^4^ (mg/kg/d)	SF_ing_^4^ (mg/kg/d)^−1^	SF_der_^4^ (mg/kg/d)^−1^
Sb	Yes	5	0.021	1.8 × 10^–3^	4.0 × 10^–4^	8.4 × 10^–6^	–	–
As	Yes	10	0.059	1 × 10^–3^	3.0 × 10^–4^	1.7 × 10^–5^	1.50	25.42
Mn	No	1	0.06	1 × 10^–3^	2.0 × 10^–2^	8.0 × 10^–3^	–	–
Pb	Yes	5	0.117	1 × 10^–4^	1.4 × 10^–3^	0.42 × 10^–3^	8.5 × 10^–3^	0.073
Zn	No	1	0.20	6 × 10^–4^	0.3	6.0 × 10^–2^	–	–
Hg	Yes	40	0.03	1 × 10^–3^	3 × 10^–4^	9.0 × 10^–6^	–	–
Cd	Yes	30	0.05	1 × 10^–3^	0.5 × 10^–3^	0.5 × 10^–5^	0.38	7.6

T: biological toxicity factor; ABS: absorption factor of stomach and intestines; KP: dermal permeability coefficient; RfD: reference doses; SF: the slope factors; *(Sharifi et al.^29^; Wen et al.^25^) **(Xiao et al.^26^; Guo et al.^11^) ‘–‘: No defined ^†^ (Mukherjee et al., 2019).

The total potential non-carcinogenic risk (HI) is evaluated using the hazard quotient (HQ) for non-carcinogenic metals. When HQ or HI > 1, the adverse effects on human health should be considered ^28^.

$$ {\text{HQ}}_{i} = \left( {{\text{ADD}}_{{{\text{ing}}}}^{i} /RfD_{{{\text{ing}}}}^{i} } \right) + \left( {{\text{ADD}}_{{{\text{derm}}}}^{i} /RfD_{{{\text{derm}}}}^{i} } \right) $$

$$ HI = \mathop \sum \limits_{i = 1}^{n} HQ_{i} $$

${\text{HQ}}_{i}$ is the hazard quotient of metal $i$. $RfD_{ing}^{i}$ and $RfD_{{{\text{derm}}}}^{i}$ are the reference doses of metal $i$ through direct ingestion and dermal contact, respectively, and $RfD_{{{\text{derm}}}}^{i} = RfD_{ing}^{i} \times {\text{ABS}}_{i}$.

The total carcinogenic risk (TCR) was evaluated using the single health risk index (CR) for carcinogenic metals. When CR > 0.1, the cancer risk is very high; 10^–3^ < CR ≤ 0.1 indicates a high risk; 10^–4^ < CR ≤ 10^–3^ indicates a moderate risk; 10^–6^ < CR ≤ 10^–4^ indicates a low risk; and CR ≤ 10–6 indicates an ignorable risk ^26–28^..

$$ {\text{CR}}_{i} = \left( {{\text{ADD}}_{{{\text{iderm}}}}^{i} \times {\text{SF}}_{{{\text{derm}}}}^{i} } \right) + \left( {{\text{ADD}}_{{{\text{derm}}}}^{i} \times {\text{SF}}_{{{\text{derm}}}}^{i} } \right) $$

$$ {\text{TCR}} = \mathop \sum \limits_{i = 1}^{n} {\text{CR}}_{i} $$

${\text{CR}}_{i}$ is the carcinogenic risk of metal $i$; $SF_{ing}^{i}$ and $SF_{derm}^{i}$ are the slope factors of metal $i$ through direct ingestion and dermal contact, respectively; and $SF_{derm}^{i} = SF_{ing}^{i} /ABS_{i}$.

## Results

### Changes in water quality indices and heavy metal concentrations in XKS

The results of the water quality indices and heavy metal concentrations of the surface water in XKS are presented in Table 2. The pH of the water was weakly alkaline (7.17–9.08). The average CODMII, COD, BOD_5_, NH_3_–N, TP, and TN values were 1.79 mg/L (0.90–3.92 mg/L), 13.51 mg/L (9.00–16.00), 2.91 mg/L (2.00–3.60 mg/L), 0.13 mg/L (0–0.40 mg/L), 0.48 mg/L (0–4.34 mg/L), and 2.58 mg/L (0–4.34 mg/L), respectively. Compared to the class II quality standard for surface water in China, the CODMII, COD, BOD_5_ and NH_3_-N values met the quality requirements (Table 2), while the TN and TP values were far beyond the standard of class II and they even exceeded class V (Table 2).

**Table 2:** Water quality indices and heavy metal concentrations in the surface water in XKS and the relative parameters.

Parameters	Surface water				Parameters for WQI			Standards for drinking water		
	Min	Max	Ave	S.D	CSS	Weight (Wi)	relative weight	Class II	Class III	Class V
pH	7.17	9.08	8.11	0.45				6–9		
CODMII (mg/L)	0.9	3.92	1.79	0.62				4	6	15
COD (mg/L)	9	16	13.51	1.90				15	20	40
BOD_5_ (mg/L)	2	3.6	2.91	0.44				3	4	10
NH3-N (mg/L)	DBL	0.401	0.13	0.10				0.5	1	2
TP (mg/L)	DBL	4.34	0.48	0.94				0.1	0.2	0.4
TN (mg/L)	0.61	5.86	2.57	1.52				0.5	1	2
Sb (mg/L)	0.0009	5.33	1.05	1.33	20	200	0.019	0.005		
As (mg/L)	DBL	19.6	1.06	3.31	10	20	0.002	0.05	0.05	0.1
Mn (mg/L)	DBL	–	–	400	10	0.001	0.1			
Pb (mg/L)	DBL	–	–	10	20	0.002	0.01	0.05	0.1	
Zn (mg/L)	DBL	0.44	0.013	0.074	3000	1	9.57E–05	1	1	2
Hg (mg/L)	DBL	0.0052	0.00084	0.00111	6	10,000	0.957	0.00005	0.0001	0.001
Cd (mg/L)	DBL	0.0036	0.00013	0.00061	3	200	0.019	0.005	0.005	0.01

The concentrations of Sb, As, Mn, Pb, Zn, Hg, and Cd in the surface water ranged from 0.0009 to 5.33 mg/L, BDL to 19.60 mg/L, ~ DBL, ~ DBL, DBL to 0.44 mg/L, DBL to 0.0053 mg/L, and DBL to 0.0036 mg/L, respectively. The mean concentrations of Sb (1.05 mg/L), As (1.06 mg/L), and Hg (0.00084 mg/L) were higher than the class III limits of the surface water quality standard, and some were even beyond the class V limits, while Zn (0.01 mg/L) and Cd (0.00013 mg/L) met the standards (Table 2). Compared with previous studies on surface water in XKS, the concentration of Sb and As is higher than that reported by Zhu Jing et al. (2009) (Sb: 5.93–7.56 mg/L, As: 0.0011–0.0073 mg/L) and Fu et al. (2016) (Sb: DBL ~ 0.16 mg/L, As: DBL ~ 0.01 mg/L), but lower than Guo et al.^12^ (Sb: 0.016 ~ 38.29 mg/L, As: 0 ~ 0.50 mg/L), Guo et al.^11^ Sb: 0.005 ~ 309 mg/L, As: 0 ~ 18.39 mg/L). Obviously, the surface water pollution in XKS has been aggravated with years of mining activity. some comprehensive treatment measures in recent years can effectively curb the aggravation of pollution, but they cannot completely eliminate the pollution.

**Figure 2:**
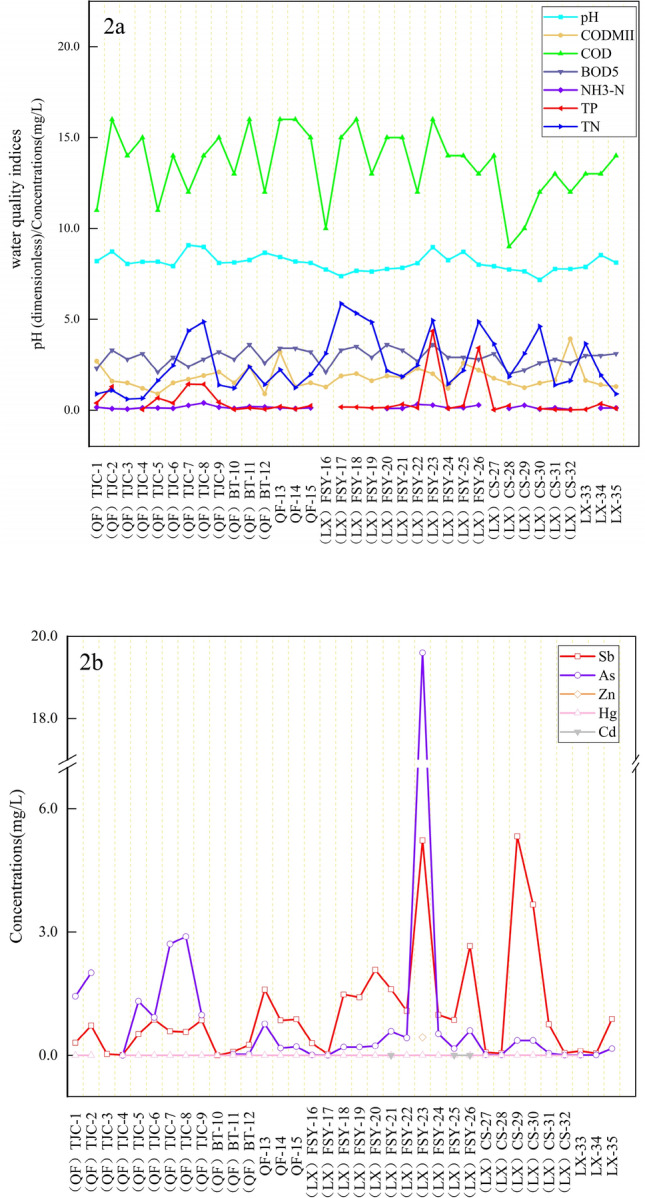
Distribution of water quality indices and heavy metal concentrations at the different sampling points in XKS.

Pearson correlation analysis (Table 3) of the metals and related water quality indexes revealed that Sb was significantly positively correlated with As (*r* = 0.707, *P* < 0.01) and Hg (*r* = 0.536, *P* < 0.01), and TN was positively correlated with Sb in the surface water (*r* = 0.391, *P* < 0.01). The pH-As (*r* = 0.643, *P* < 0.01) and TP–Sb (*r* = 0.785, *P* < 0.05) were significantly positively correlated. From the perspective of the spatial distribution, the variations in the surface water quality indexes (Fig. 2a) were also consistent with the metal concentrations (Fig. 2b). However, the heavy metal concentrations and types were different in the rivers due to the distribution of the smelters and waste dumps. The As content of the watershed of Qingfeng River was significantly higher than the Sb content, while the opposite was observed in the Lianxi River watershed. The metal concentrations of the following samples were high: samples 5 to 9 from the lower reaches of Tanjiachong stream close to the waste rock pile; samples 18 to 26 from the middle to lower reaches of Feishuiyan stream where it flows past a smelting plant; and samples 29 to 30 from the left tributaries of the upper reaches of Chuanshan Stream adjacent to the smelter and the waste rock pile.

**Table 3:** Pearson correlation coefficients of the water quality indices and heavy metal concentrations in the surface water in XKS.

	pH	CODMII	COD	BOD_5_	NH_3_–N	TP	TN	Sb	As	Hg	Cd
pH	1										
CODMII	0.047	1									
COD	0.237	0.17	1								
BOD_5_	0.117	0.196	.954**	1							
NH3-N	.457*	0.045	− 0.091	-0.09	1						
TP	.457**	0.074	0.123	0.088	.562**	1					
TN	− 0.145	0.091	0.037	0.099	.647**	.422*	1				
Sb	− 0.088	-0.01	0.02	0.091	0.319	.643**	.391*	1			
As	.455**	0.062	0.225	0.225	0.353	.785**	0.288	.536**	1		
Hg	− 0.305	− 0.03	− 0.259	-0.157	0.02	0.203	0.216	.707**	0.307	1	
Cd	0.994	0.904	− 0.082	− 0.404	− 0.271	− 0.447	− 0.332	− 0.764	− 0.993	− 0.082	1

*At level 0.05 (two-tailed), the correlation was significant.

**At level 0.01 (two–tailed), the correlation was significant.

### Heavy metal pollution assessment

**Figure 3:**
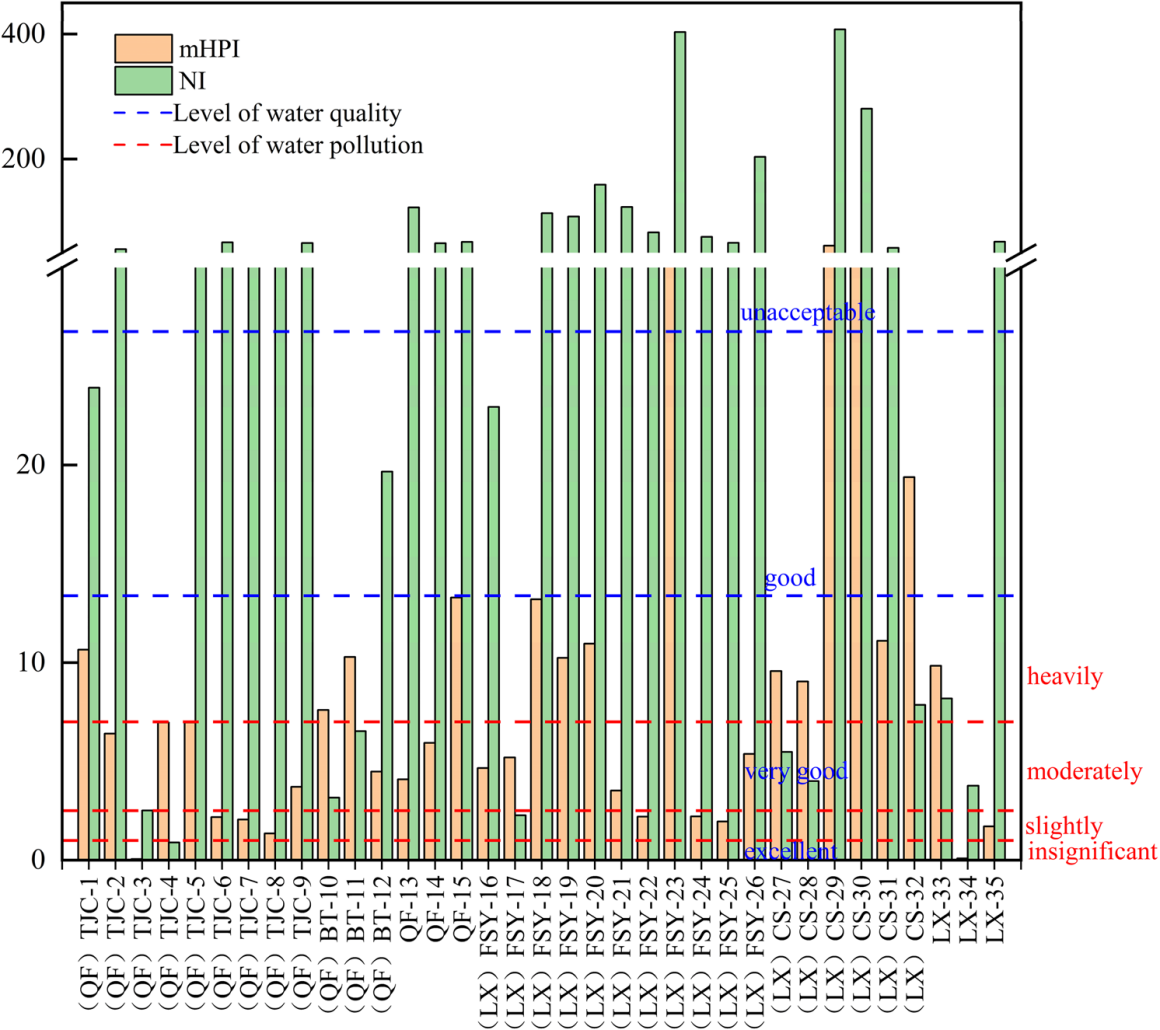
Heavy metal pollution of surface water in XKS based on the NI and m-HPI.

The NI (the Nemerow index) and m-HPI methods were used to evaluate the water pollution by heavy metals in XKS (Fig. 3). Based on the m-HPI water quality scale, the water quality of 2.85–88.57% of the sampling sites in the study area was good to very good, and only 8.57% of the sites had the worst water quality. The NI values ranged from 0.90 to 407.49, with an average of 80.80. According to the NI water quality classification standard, about 5.70% of the sampling sites were insignificantly to lightly polluted, and 7.14% and 87.16% were moderately and severely polluted, respectively. The pollution level assessed using the NI method was more severe than that assessed using the m-HPI method (Fig. 3), which is contrary to the results for groundwater quality in Indian using the same methods^4^. In the evaluation using the NI, the influence of the maximum value will be overemphasized and the other factors will be artificially ignored^30^. Sb and As are the most important pollutants in the area. Several studies have shown that Sb contents of food, water and soil are several times greater than their As contents^11^. Therefore, the impact of the high Sb content may be exaggerated, while those of other trace metals may be weaker. However, both results indicate that the quality of samples 23, 29, and 30 was the worst, and the pollution level was far higher than that of the other samples. It is necessary to be alert to the release of metals in these areas.

### Ecological risk assessment

**Figure 4:**
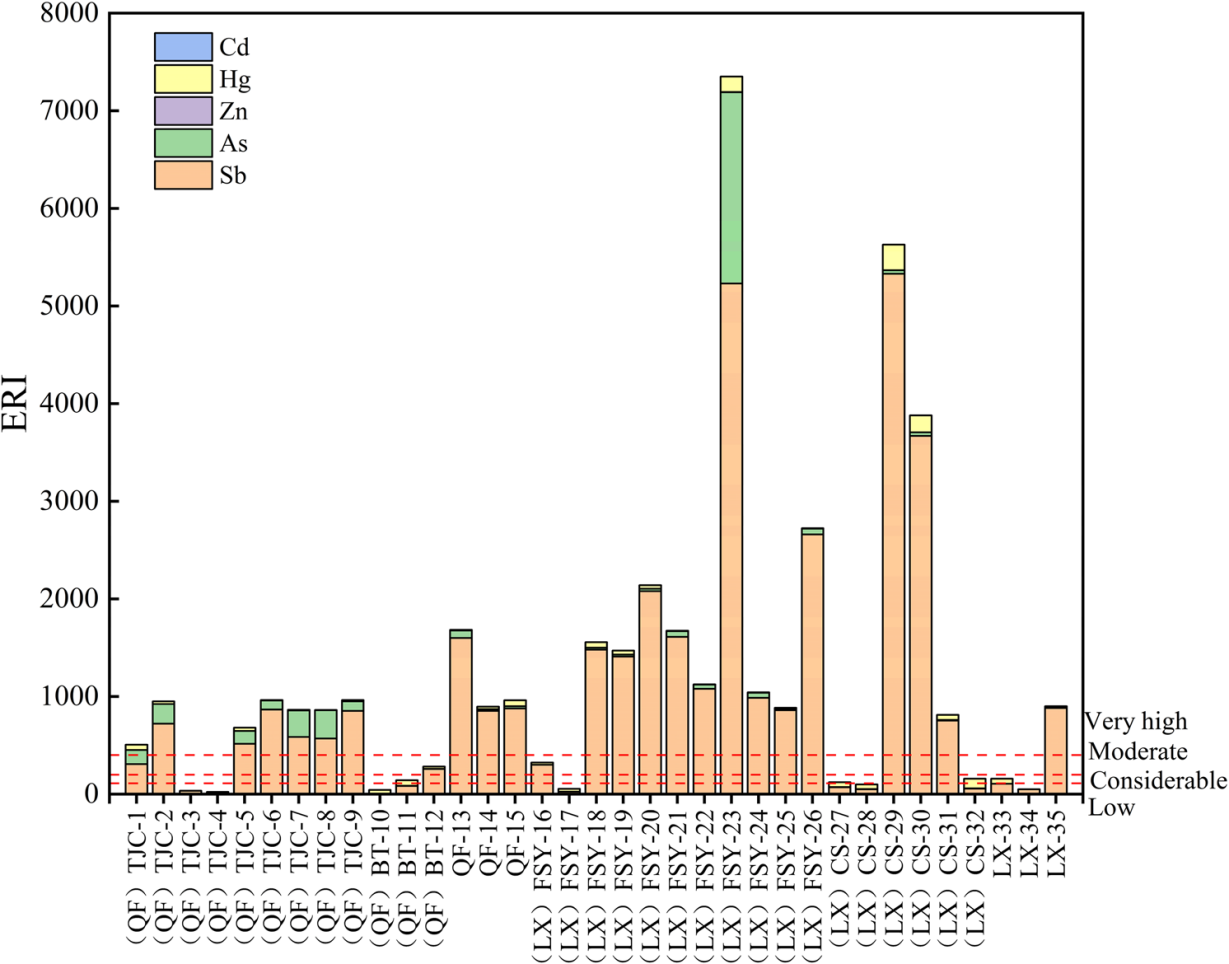
Ecological risks of the heavy metal in the surface water in XKS.

The ERI values in XKS ranged from 22.69 to 7351.20, with an average of 1200.40. In the study area, 17.14% of the samples had a low ecological risk, 11.43–65.71% of the samples had a moderate to high risk, and more than 50% had a very high risk (Fig. 4). Sb and As were the main pollutants, followed was Hg. The biotoxicity factors of Hg and Cd were the highest, with contributions of 43.48% and 32.61%, respectively, so they are the main risk elements. The detection rates of Hg and Cd in the water were 91.43% and 8.57%, respectively. The Cd contents in the water were low in most places, but at sampling sites 23, 29, and 33, Cd posed a threat to the ecology, with ERI values of ≥ 110. The contributions of As, Sb, and Pb were 10.87%, 5.43%, and 5.43%, respectively, and their detection rates in the surface water were 94.29%, 100%, and 0.00%, respectively. In addition, 71.43% and 17.14% of the samples had a moderate or greater ecological risk related to Sb and As (ERI ≥ 110), respectively. Studies have shown that Sb and As in surface water are toxic to organisms and humans at a certain level^11,31^. In particularly, near the antimony mining area, the acute and chronic potential risk levels of Sb in the surface water reached 35.00% and 40%, respectively, posing a serious threat to the water ecosystem^31^.

### Health risk assessment

**Figure 5:**
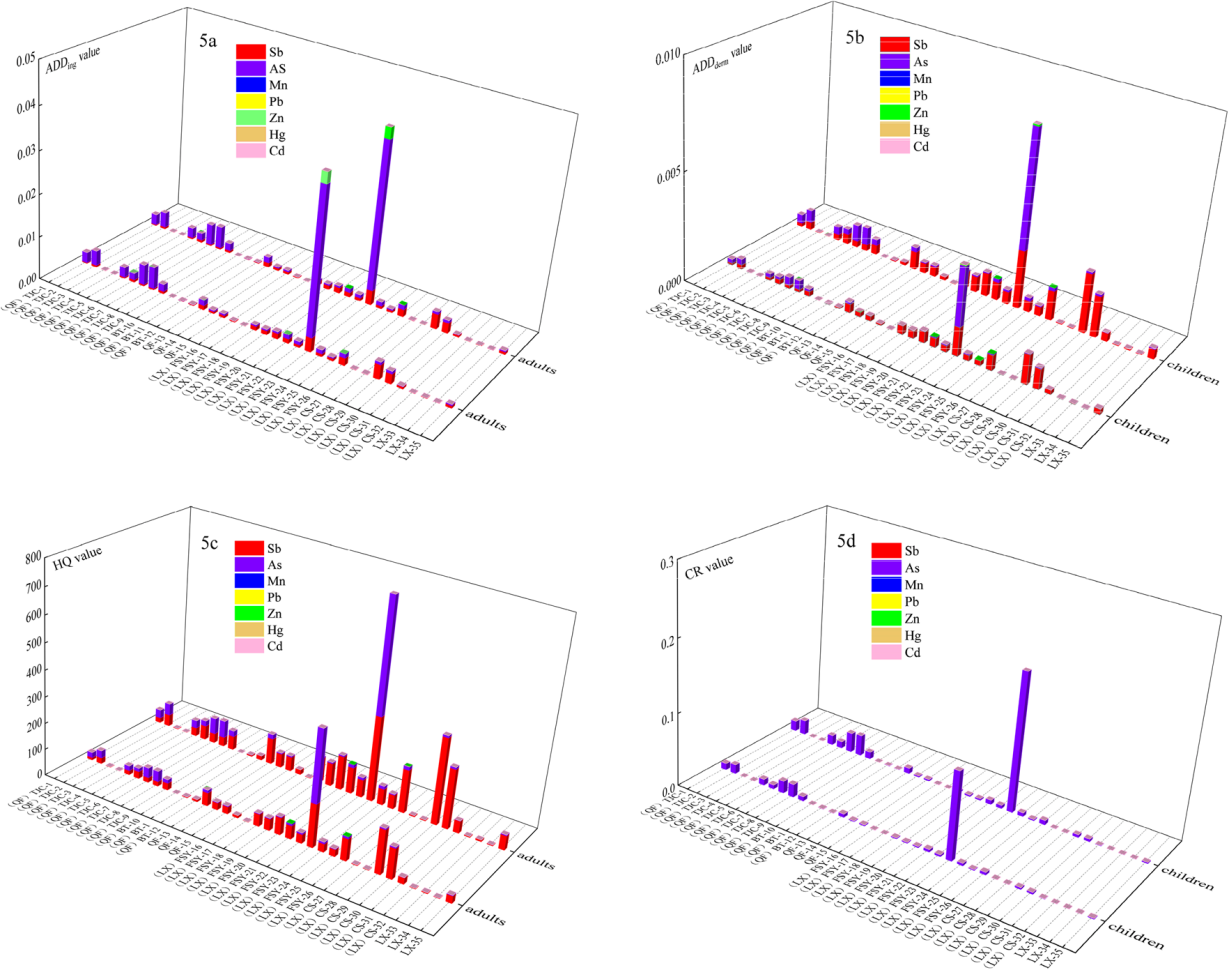
(**a** and **b**) ADD, (**c**) HI and (**d**) CR for metals in the surface water in XKS through ingestion and skin contacting for adults and children.

The non-carcinogenic (HI) and carcinogenic (CR) health risks to residents were calculated based on the daily intake doses (AAD) of the heavy metals in the surface water in XKS under two exposure pathways: direct ingestion (AAD_ing_) and dermal contact (AAD_derm_). In terms of the daily exposure dose (Fig. 5a and b), the absorption of Sb and As was the main factor for all of the people in the area under the two pathways. The Sb AAD_ing_ values for adults and children were 6.61 × 10^–4^ mg^.^kg^−1^d^−1^ and 6.88 × 10^–4^ mg^.^kg^−1^d^−1^, respectively, and both were higher than the threshold values for the oral reference dose (4 × 10^–4^ mg^.^kg^−1^d^−1^) and the average ADD_derm_ value for adults (2.63 × 10^–4^ mg^.^kg^−1^d^−1^) and children (5.3 × 1010^–4^ mg^.^kg^−1^d^−1^). The ADD_ing_ values of As for the two groups were 18.42 × 10^–4^ mg^.^kg^−1^d^−1^ and 19.16 × 10^–4^ mg^.^kg^−1^d^−1^, higher than the daily As intake dose of drinking water (1.05 × 10^–4^ mg^.^kg^−1^d^−1^) reported in previous studies^11^, and the ADD_derm_ of As for adults (1.47 × 10^–4^ mg^.^kg^−1^d^−1^) and children (3.01 × 10^–4^ mg^.^kg^−1^d^−1^). Thus, the intake dose through direct ingestion was higher than that through skin contact. It should be noted that the absorption of Zn for sample 23 through direct ingestion for adults and children was relatively higher. For the different groups, the daily intake of the metals through skin was higher for children than for adults, while the intakes through direct ingestion was comparable for both groups. However, under both exposure pathways, the ADD of As was higher than that of Sb in the Qingfeng River, and the opposite occurred in the Lianxi River Basin.

In this study, the total non-carcinogenic risk (HI) of the heavy metals in the surface water was represented by the hazard quotient (HQ). When the HQ is greater than 1, there is a health hazard to humans. The results revealed that the average HQs of Sb, As, Zn, Cd, and Hg for adults were 32.91, 14.77, 0.0003, 0.015, and 0.004, respectively, and the HI was 47.70. The corresponding mean HQs for children were 65.94, 24.12, 0.0003, 0.029, and 0.008, and the HI value was 90.10. This indicates that the residents in the XKS area suffer considerable chronic non-carcinogenic health risks. Sb is the greatest contributor, followed by As, both of which are higher than previously reported (HQ_Sb_ and HQ_As_ of 27.0 ± 21.3 and 3.97 ± 1.39, respectively)^11^. Real case investigations have demonstrated the toxic effects of mixed Sb and As pollution on humans^32^. Sb and As are ubiquitous and persistent in the environment, and the main organs harmed by their toxicity include the skin, the liver, kidney, and respiratory system^33,34^. According to the comparison shown in Fig. 5c, we conclude that the heavy metals pose a higher risk to children.

The CR values of As and Cd for the different groups were calculated using the slope factor (Fig. 5d). The results revealed that the risk from Cd exposure was relatively low for the entire population, and the largest contributor to the total carcinogenic risk was As. For adults and children, the average CR values for As were 6.49 × 10^–3^ and 1.05 × 10^–2^, respectively, which are higher than previously reported^11^, and are above the threshold value of 1 × 10^–4^, indicating a high carcinogenic risk. Long-term contact with water containing As may lead to potential carcinogenic effects and diseases such as hypertension and neuropathy^35^, especially in children. Children are a sensitive part of the population, and they have a higher risk^26^. However, As and Sb have similar internal exposure characteristics, and their concentrations are correlated with age and gender^11^. Males and middle-aged people have higher As levels than females and the elderly, which is mainly attributed to the fact that men are mainly exposed through labor related to Sb ore^11^.

## Conclusions

In this study, the characteristics of the water quality and metal concentrations in the surface water in XKS were analyzed. The results show that the concentrations of TN, TP, Sb, As and Hg in XKS surface water far exceed the surface water quality standards of China, and significantly correlated with each other. The pollution assessment revealed that the results obtained using the NI were more serious than those obtained using m-HPI, the assessment method to accurately assess metal pollution should be chosen comprehensively. The ecological risk assessment indicated that Sb and As were the main toxicity factors, followed by Hg. Regarding the health risk assessment, Sb and As were the major threats to human health, especially children's health. It should be noted that the concentration of As in the Qingfeng River Basin was higher than the Sb concentration in the XKS area, while the opposite occurred in the Lianxi River. Thus, rivers in different areas varies in pollutants and pollution levels which pose a greater risk to human health and the ecological balance.. Based on this, it is suggested to carry out more detailed investigation on the small scale and improve treatment and restoration measures, for controlling the release of pollution elements from their sources and tackle the problems directly beyond the general governance in rivers.

## Data Availability

The datasets used during the current study are available from the author Qing Xie (xieqing314934@outlook.com) on reasonable request.
